# CD38-specific nanobodies allow *in vivo* imaging of multiple myeloma under daratumumab therapy

**DOI:** 10.3389/fimmu.2022.1010270

**Published:** 2022-10-27

**Authors:** Luca Julius Pape, Julia Hambach, Anna Josephine Gebhardt, Björn Rissiek, Tobias Stähler, Natalie Tode, Cerusch Khan, Katja Weisel, Gerhard Adam, Friedrich Koch-Nolte, Peter Bannas

**Affiliations:** ^1^ Department of Diagnostic and Interventional Radiology and Nuclear Medicine, University Medical Center Hamburg-Eppendorf, Hamburg, Germany; ^2^ Institute of Immunology, University Medical Center Hamburg-Eppendorf, Hamburg, Germany; ^3^ Department of Neurology, University Medical Center Hamburg-Eppendorf, Hamburg, Germany; ^4^ Department of Oncology, Hematology and Bone Marrow Transplantation, University Medical Center Hamburg-Eppendorf, Hamburg, Germany

**Keywords:** CD38, daratumumab, multiple myeloma, nanobody, fluorescence imaging, flow cytometry

## Abstract

**Rationale:**

Recent studies have demonstrated the feasibility of CD38-specific antibody constructs for *in vivo* imaging of multiple myeloma. However, detecting multiple myeloma in daratumumab-pretreated patients remains difficult due to overlapping binding epitopes of the CD38-specific imaging antibody constructs and daratumumab. Therefore, the development of an alternative antibody construct targeting an epitope of CD38 distinct from that of daratumumab is needed. We report the generation of a fluorochrome-conjugated nanobody recognizing such an epitope of CD38 to detect myeloma cells under daratumumab therapy *in vitro*, *ex vivo*, and *in vivo*.

**Methods:**

We conjugated the CD38-specific nanobody JK36 to the near-infrared fluorescent dye Alexa Fluor 680. The capacity of JK36^AF680^ to bind and detect CD38-expressing cells pretreated with daratumumab was evaluated on CD38-expressing tumor cell lines *in vitro*, on primary myeloma cells from human bone marrow biopsies *ex vivo*, and in a mouse tumor model *in vivo*.

**Results:**

Fluorochrome-labeled nanobody JK36^AF680^ showed specific binding to CD38-expressing myeloma cells pretreated with daratumumab *in vitro* and *ex vivo* and allowed for specific imaging of CD38-expressing xenografts in daratumumab-pretreated mice *in vivo*.

**Conclusions:**

Our study demonstrates that a nanobody recognizing a distinct, non-overlapping epitope of CD38 allows the specific detection of myeloma cells under daratumumab therapy *in vitro*, *ex vivo*, and *in vivo.*

## Introduction

CD38 is a major target for the therapy of multiple myeloma (MM). Daratumumab is a CD38-specific monoclonal antibody with high efficacy as monotherapy or combination therapy for relapsed and newly diagnosed multiple myeloma (1–4). Daratumumab therapy has been integrated into international treatment guidelines and has become the standard of care (5). Reliable and accurate assessment of treatment response is needed even in the presence of therapeutic daratumumab plasma levels. Unfortunately, this presents a diagnostic challenge since daratumumab interferes both with flow cytometry (6–9) as well as with free light chain assays (10) and serum immunofixation electrophoresis (11).

Myeloma manifestations can alternatively be detected *in vivo* by cross-sectional imaging techniques such as whole-body computed tomography, magnetic resonance imaging (12), and ^18^F-FDG-positron emission tomography (PET) (13–15). These imaging techniques detect medullary and extramedullary myeloma lesions with high sensitivity (16). However, these techniques do not allow monitoring of CD38 expression or prediction of susceptibility to daratumumab treatment since they are not antigen-specific to CD38.

Immuno-positron emission tomography using radiolabeled CD38-specific antibodies overcomes this challenge, thereby enabling the detection and visualization of CD38-expressing myeloma cells *in vivo* (17–20). Unfortunately, detecting multiple myeloma in daratumumab-pretreated patients remains difficult due to overlapping binding epitopes of currently available CD38-specific imaging antibody constructs and daratumumab. Therefore, the development of alternative antibody constructs targeting a different epitope of CD38 is needed.

**Figure 1:**
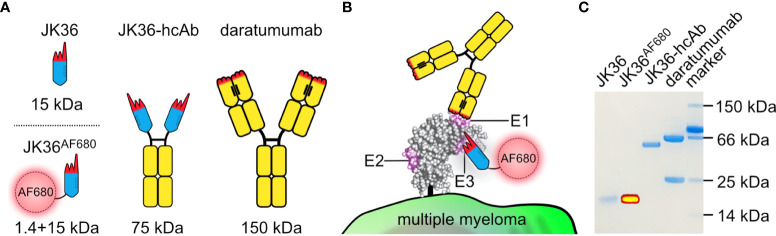
Structure, binding sites, and purity of JK36^AF680^ nanobody, JK36 heavy chain antibody, and daratumumab. **(A)** Comparison of different antibody constructs targeting CD38. The framework of single-domain antibody (nanobody) JK36 is indicated in blue with the CDR-regions indicated in red. Heavy-chain antibody JK36-hcAb consists of two heavy chains each containing nanobody JK36 fused to the hinge (black), CH2, and CH3 domains (yellow) of human IgG1. The conventional human IgG1 mAb daratumumab is also indicated in black and yellow. The hydrophobic interface between the two variable domains of daratumumab is replaced by a corresponding hydrophilic region in JK36, accounting for the excellent solubility of this VHH domain in absence of a light chain. **(B)** Daratumumab (epitope E1) and JK36^AF680^ (epitope E3) recognize two distinct, non-overlapping epitopes (E1 and E3) of CD38. Epitope 2 (E2) is recognized by nanobody JK2 (not shown) and was used in our study for control staining of CD38. **(C)** 1 µg of purified JK36^AF680^, JK36-hcAb, and daratumumab were size-fractionated by SDS-PAGE and visualized by Coomassie staining. Superimposed fluorescent signals (yellow) were recorded using a near-infrared fluorescence *in vivo* imaging system.

Nanobodies are single variable immunoglobulin domains derived from camelid heavy-chain antibodies (21). CD38-specific nanobodies can be used either for the treatment of multiple myeloma by generation of nanobody-based heavy-chain antibodies (hcAbs) (22–26), nanobody-based CARs (27), and nanobody-based BiKEs (28), or for *in vivo* imaging of multiple myeloma (29). Their small molecular size (**Figures 1A, B**), low immunogenicity, and ease of formatting make them ideally suited for *in vivo* imaging purposes (21, 30–33).

The aim of our study was to generate a fluorochrome-conjugated nanobody recognizing an epitope of CD38 distinct from that of daratumumab to detect tumor cells under daratumumab therapy *in vitro*, *ex vivo*, and *in vivo*.

## Materials and methods

### Cell lines

Three human multiple myeloma cell lines (LP-1, U266, RPMI-8226), two human Burkitt lymphoma cell lines (Daudi and CA-46), and a murine B cell lymphoma cell line (YAC-1) were obtained from the German Collection of Microorganisms and Cell Culture (DSMZ, Braunschweig, Germany). Human cell lines were chosen due to their uniform expression of CD38. Stable expression of *Photinus pyralis* luciferase (Promega, Madison, WI, USA) in Daudi luc, CA-46 luc, YAC-1 luc, and LP-1 luc cell lines was achieved by lentiviral transduction as described previously (25, 34). YAC-1 luc cells were stably transfected with human CD38 using the expression vector pEF-DEST51 (29), yielding YAC-1 CD38+ cells. Untransfected YAC-1 cells served as negative controls.

### Production and labeling of antibody constructs

Human CD38-specific nanobody JK36 was generated from an immunized llama as described previously (29, 35). His/myc-tagged nanobody JK36 was produced in HEK293-6E cells and purified from supernatants using immobilized metal affinity chromatography (29). Nanobody JK36 was labeled with the fluorescent dye Alexa Fluor 680 (JK36^AF680^) according to the manufacturer’s instructions using succinimidyl esters (Invitrogen, Karlsbad, CA, USA).

Heavy chain antibodies (hcAbs) JK36-hcAb and isotype control L-15-hcAb were generated by subcloning the coding region of nanobody JK36 and isotype control nanobody L-15 upstream of the coding region for the hinge, CH2, and CH3 domains of human IgG1 in the pCSE2.5 vector (kindly provided by Thomas Schirrmann, University of Braunschweig, Braunschweig, Germany) (24). HcAbs were produced in HEK293-6E cells and purified from supernatants by affinity chromatography using protein A sepharose (36, 37). Daratumumab (Darzalex) was purchased from Janssen-Cilag, Neuss, Germany.

The purity of antibody constructs was assessed by SDS-PAGE and InstantBlue™ Coomassie staining. Alexa Fluor 680-labeling of nanobody JK36 was controlled by imaging fluorescence levels using the IVIS-200 *in vivo* imaging device (PerkinElmer, Waltham, MA, USA).

Monoclonal antibody HIT2^PerCP/Cy5.5^ was purchased from Becton Dickinson, Franklin Lakes, NJ, USA (25).

### Flow cytometry

For CD38-expression analyses, CD38-positive cells and CD38-negative control cells were incubated with JK36^AF680^ (0,2 µl in 100 µl PBS/BSA) at 4 °C for 30 min. Cells were washed twice and analyzed using a FACS Canto II flow cytometer and FlowJo software (Becton Dickinson, Franklin Lakes, NJ, USA).

CD38 pretreatment (i.e., cross-blockade) analyses were performed by pre-incubating cells with an excess (200 nM) of daratumumab or JK36-hcAb at 4 °C for 30 min. Cells were then incubated with nanobody JK36^AF680^ (1:500 in PBS/BSA) to detect unblocked epitopes using flow cytometry.

### Biolayer interferometry

The extracellular domain of human CD38 (aa 46-300) was produced, purified, and biotinylated as described previously (22, 28). Biolayer interferometry analysis was performed at 20°C in kinetic buffer (PBS containing 1% bovine serum albumin, and 0.005% (v/v) polysorbate 80 (Tween 80, Sigma-Aldrich, St. Louis, MO, USA)). For individual antibodies, concentrations of 500 nM per antibody were used while antibody combinations were analyzed at a concentration of 250 nM per antibody to allow for complete saturation of CD38. Biolayer interferometry measurements were carried out using a BLItz system (FortéBio, Fremont, CA, USA).

Streptavidin-coated biosensors were placed in wells containing only kinetic buffer for 30 seconds to establish a baseline signal. Sensors were then transferred to wells containing the biotinylated extracellular domain of human CD38 for 90 seconds to allow for association of CD38 to the sensor. This was followed by 30 seconds of dissociation in kinetic buffer. CD38-coated sensors were subsequently dipped into wells containing the blocking antibody daratumumab for 90 seconds to saturate epitope E1. Sensors were then moved to wells containing both the blocking antibody daratumumab and a second antibody (daratumumab, JK36-hcAb, or L-15-hcAb) for 90 seconds. This permitted assessment of binding of the second antibody to CD38 in the presence of the blocking antibody as opposed to solely evaluating binding to CD38. This protocol considers any possible inhibitory effects that excess unbound daratumumab might have on secondary antibody binding. A final washing step in kinetic buffer was performed for 90 seconds to monitor the dissociation of CD38-bound antibodies. The resulting dataset was plotted using GraphPad Prism 9.3.1 (GraphPad Software, CA, USA).

### Fluorescence microscopy

YAC-1 CD38+ cells were incubated with an excess of daratumumab (3 µg/100 µl), JK36-hcAb (3 µg/100 µl), or no blocking agent at 4 °C for 20 min. Cells were washed once with PBS/0,02% BSA. Cells were then resuspended in 100 µl PBS/BSA containing JK36^AF680^ at a dilution of 1:500 and diamidino-phenylindole (DAPI) at a dilution of 1:5000 (v/v) and incubated at 4 °C for 20 min to stain CD38 and nuclei, respectively. Stained cells were washed twice before resuspension in 100 µl of PBS/BSA. Twenty µl of each sample was subsequently placed on a glass microscopy slide without addition of a fixative agent. Cell samples were then captured using a Zeiss Axio Observer microscope with a 40x EC Plan-Apochromat Oil lens. Microscopic images were analyzed using ZEN Pro 3.4 software (Zeiss, Oberkochen, Germany) and Affinity Designer 1.10.5 (Serif, Nottingham, UK).

### Flow cytometric analysis of primary human bone marrow samples

Aspiration of fresh bone marrow was approved by the Institutional Review Board (PV5505). Fresh bone marrow aspirates were collected from nine newly diagnosed, untreated multiple myeloma patients. Ficoll-Paque density gradient centrifugation (Sigma-Aldrich, St. Louis, MO, USA) was carried out to isolate bone marrow mononuclear cells (BM-MNCs). Remaining erythrocytes were depleted by resuspending the resulting cell pellet in red cell lysis buffer (NH4Cl + KHCO3 + EDTA).

BM-MNCs were stained using PacO and a panel of fluorochrome-labeled antibodies targeting CD19, CD38, CD39, CD45, CD55, CD56, CD59, CD138, CD229, CD319 and analyzed by flow cytometry to determine the degree of bone marrow infiltration with malignant plasma cells (27). Multiple myeloma cells were identified by high expression of CD38.

Blocking assays were carried out by incubating BM-MNCs without (positive control) or with 100 nM of daratumumab or JK36-hcAb in PBS/BSA at 4 °C for 30 min. After one washing step, detection nanobody JK36^AF680^ was added for another 30 min. Mean fluorescence intensities (MFI) of bound nanobody JK36^AF680^ were assessed by flow cytometry. Relative fluorescence intensities (%) of multiple myeloma cells labeled with nanobody JK36^AF680^ were calculated as follows:


$$relative\;fluorescence\;intensity_{sample}\;[\%]=\frac{MFI\;{\left(AF680\right)}_{sample}}{MFI\;{\left(AF680\right)}_{positive\;control}}x100\%$$

Significant differences in relative fluorescence intensities were calculated by using one-way ANOVA (GraphPad Prism 9.3.1).

### *In vivo* and *ex vivo* imaging

Animal experiments were approved by the local animal welfare commission (N069/2018). Six-week-old female NMRI Foxn1nu mice were acquired from Charles River (Charles River Laboratories, Sulzfeld, Germany) and kept in their cages for two weeks. Eight-week-old mice were kept on an alfalfa-free diet for one week prior to *in vivo* imaging experiments to reduce autofluorescence of the intestine (38).

Tumors were generated by subcutaneously injecting mice with 1x10^7^ CD38-negative YAC-1 cells on the left shoulder and 5x10^6^ CD38-positive YAC-1 cells on the right shoulder in 0.2 µl of 50% RPMI and 50% Matrigel (Becton Dickinson, Franklin Lakes, NJ, USA). This established murine lymphoma model (29) was chosen because the reliable subcutaneous growth of YAC-1 cells allows for intraindividual comparison of antigen-positive and antigen-negative tumors using near-infrared fluorescence imaging. This would not have been feasible using a disseminated human myeloma model. Numbers of injected cells were adjusted to account for the slightly slower growth rate of CD38-negative vs. CD38-positive YAC-1 cells. Tumor locations at shoulder level were chosen to maximize the distance to the kidneys and liver, which had shown high fluorescence signals in previous studies (29, 33). After six days, mice (total, n=30) were intravenously injected with either 100 µl of isotonic saline containing 500 µg of daratumumab (n=10 mice) (Janssen Biotech, Horsham, PA, USA), 250 µg of JK36-hcAb (n=10 mice), or no blocking agent (n=10 mice). After 24 hours, mice were intravenously injected with 50 µg of JK36^AF680^.

*In vivo* near-infrared fluorescence imaging was performed under isoflurane anesthesia before and 2, 4, 6, 12, and 24 hours after injection of JK36^AF680^ using a small animal imaging system (IVIS-200, PerkinElmer, Waltham, MA, USA) (29). After qualitative imaging *in vivo*, quantitative off-line analyses were performed by placing ROIs around CD38-positive tumors, CD38-negative tumors (negative control), and the hind limb (background signal). Total radiant efficiency was determined with Living Image 4.2 software (PerkinElmer) and the background value was subtracted. Tumor-to-background ratios were calculated by dividing the tumor uptake value by the background value. Radiant efficiencies and tumor-to-background ratios were compared between treatment groups using a mixed-effect analysis with Tukey’s multiple comparison test (GraphPad Prism 9.3.1).

For *ex vivo* validation of *in vivo* measurements, three mice from each treatment group were sacrificed six hours after injection. Tumors and organs (muscle, spleen, lungs, liver, kidneys, stomach, intestine) were dissected and imaged *ex vivo* with the IVIS-200 system.

### *Ex vivo* flow cytometric analyses of cells from explanted subcutaneous tumors

Single cell suspensions from explanted and dissected CD38-positive and CD38-negative tumors were generated by passage through a cell strainer with a pore size of 70 µm (Corning Life Sciences, Corning, NY, USA).

Cell surface levels of CD38 on resuspended tumor cells were determined using hcAb JK2 rabbit IgG, which binds a third epitope of CD38 distinct from that of both daratumumab and JK36-hcAb. Bound JK2-hcAb was detected with a rabbit IgG-specific, R-phycoerythrin-conjugated secondary antibody (Cat.-No. 711-116-152, Jackson ImmunoResearch, Ely, UK) (29).

Quantification of *in vivo* injected and tumor-bound blocking antibodies daratumumab and JK36-hcAb was performed by labeling resuspended tumor cells with an R-phycoerythrin-conjugated human IgG-specific secondary antibody (Cat.-No. 709-116-149, Jackson ImmunoResearch). Mean fluorescence intensities were obtained by flow cytometry to determine and compare binding (i.e. blocking efficiencies) of daratumumab and JK36-hcAb.

Quantification of *in vivo* injected and tumor-bound imaging nanobody JK36^AF680^ was performed by flow cytometric analysis of resuspended tumor cells. Mean fluorescence intensities of CD38-labeling efficiencies with JK36^AF680^ were compared between groups of mice pre-treated with either daratumumab, JK36-hcAb, or saline.

To determine the maximum achievable labeling efficiency of CD38 with JK36^AF680^, cells were labeled *ex vivo* with saturating doses (100 nM) of JK36^AF680^.

CD38-positive and CD38-negative YAC-1 luc cells from cell culture were incubated as described above and served as controls.

## Results

Purity of nanobody JK36, JK36-hcAb, and daratumumab was confirmed by SDS-PAGE analyses. Successful labeling of purified nanobody JK36 with Alexa Fluor 680 (JK36^AF680^) was verified by imaging of the SDS-PAGE gel with the *in vivo* imaging system (**Figure 1C**).

### Simultaneous binding of nanobody JK36 and daratumumab to purified CD38

**Figure 2:**
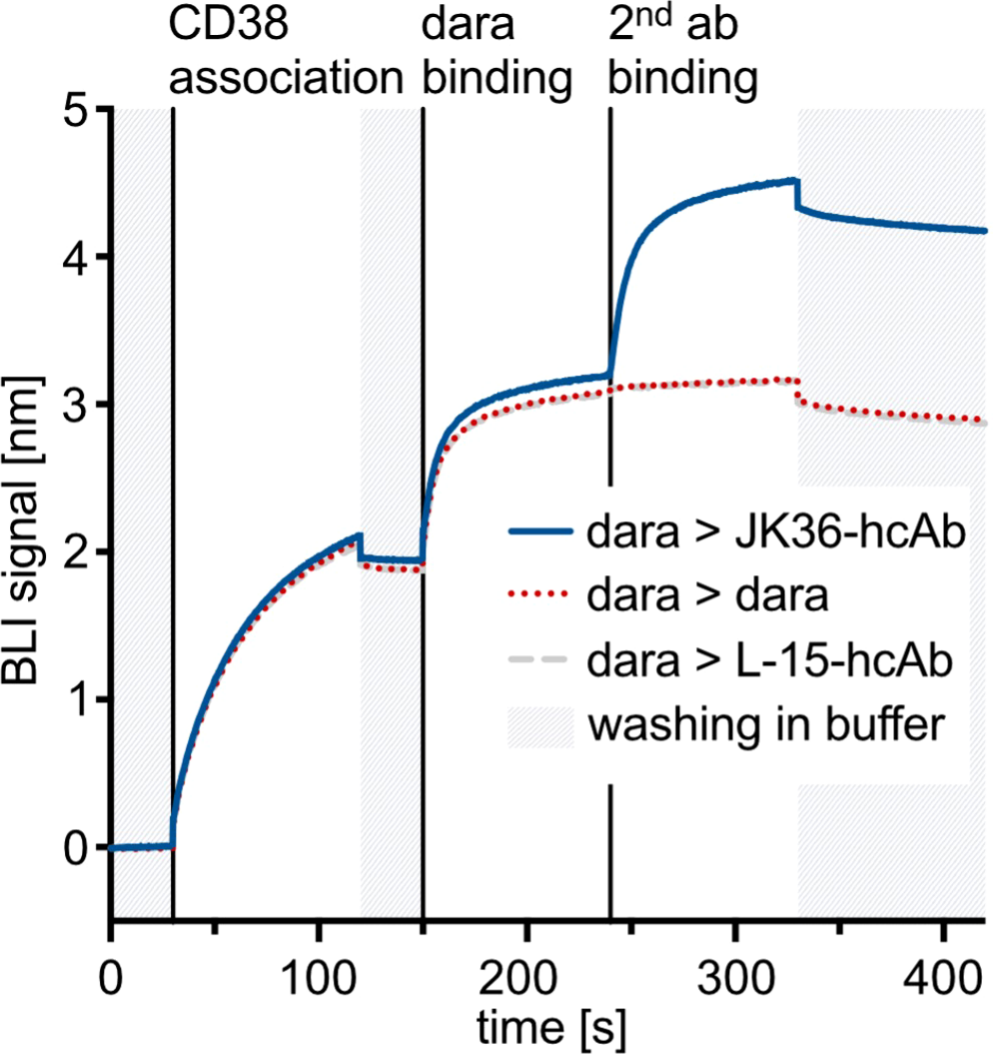
Biolayer interferometry sensograms demonstrating simultaneous binding of nanobody-based JK36-hcAb and daratumumab to purified CD38. Streptavidin-coated biosensors were used to capture the biotinylated extracellular domain of CD38. Sensors were then dipped into wells containing daratumumab in excess before being transferred to wells containing both daratumumab and secondary antibodies at the indicated time points. An increase in signal indicates binding to the sensor. Dissociation was allowed by washing in buffer and is indicated in grey.

Biolayer interferometry was used to determine whether nanobody JK36 and daratumumab bind simultaneously to CD38. After binding of biotinylated CD38 to a streptavidin-coated sensor, blocking antibody daratumumab and secondary antibodies (JK36-hcAb, daratumumab, or isotype control L-15-hcAb) were added sequentially (**Figure 2**).

The results show an increase in signal intensities upon the addition of the blocking antibody daratumumab. After adding the secondary antibody JK36-hcAb, a strong increase in signal intensity was observed, indicating simultaneous binding of daratumumab and JK36-hcAb. In contrast, adding daratumumab as a secondary antibody did not lead to further signal increase, indicating saturated binding of daratumumab as the primary antibody to CD38. Isotype control L-15-hcAb also did not lead to a signal increase, indicating specific binding of JK36-hcAb to CD38.

These results demonstrate that nanobody JK36 and daratumumab bind independently and simultaneously to CD38.

### Specific binding of nanobody JK36^AF680^ to daratumumab-pretreated myeloma cells *in vitro* and *ex vivo*

**Figure 3:**
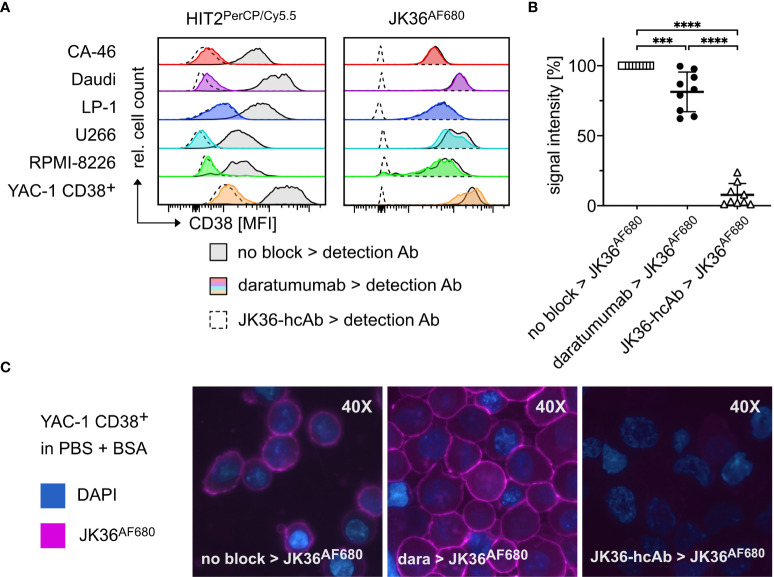
Specific binding of nanobody JK36^AF680^ to daratumumab-pretreated myeloma cells *in vitro* and *ex vivo*. **(A)** FACS analyses of CD38+ CA-46, Daudi, LP-1, U266, RPMI-8226, and YAC-1 CD38+ cells. Cells were saturated with daratumumab before detection of CD38 with the CD38-specific diagnostic antibody HIT2^PerCP/Cy5.5^ (left) or the CD38-specific nanobody JK36^AF680^ (right). Cells saturated with JK36-hcAb served as negative controls since previous experiments had shown complete blocking of both JK36^AF680^ and HIT2^PerCP/Cy5.5^ binding after incubation with JK36-hcAb. Untreated cells were used as positive controls and indicate CD38 expression of the cell line. **(B)** Bone marrow aspirates of human multiple myeloma patients were pre-incubated without (open rectangles, positive control) or with saturating doses of daratumumab (black circles) or JK36-hcAb (open triangles, negative control). CD38 was then labeled using JK36^AF680^ and cells were analyzed by flow cytometry for comparison of epitope blocking by daratumumab and JK36-hcAb. Signal intensities were compared using one-way ANOVA with Tukey’s multiple comparisons test (****= p<0.0001, ***=p<0.001). Depicted are means ± SD. Results are representative of nine independent experiments. **(C)** Fluorescence microscopy analyses of YAC-1 CD38+ cells. Cells were incubated with PBS/BSA (left panel), daratumumab (middle panel), or JK36-hcAb (right panel) before the addition of JK36^AF680^. DAPI was used to stain nuclei. Results are representative of three independent experiments.

*In vitro* binding of nanobody JK36^AF680^ to daratumumab-pretreated cells was assessed using five human (LP-1, U266, RPMI-8226, CA46, Daudi) lymphoma cell lines, and one murine lymphoma cell line transfected with human CD38 (YAC-1 CD38+) (**Figure 3A**). CD38-specific monoclonal antibody HIT2^PerCP/Cy5.5^ was used as control. Cells were incubated without or with saturating doses of daratumumab and then stained with nanobody JK36^AF680^ or monoclonal antibody HIT2^PerCP/Cy5.5^ followed by flow cytometry.

The results show that both nanobody JK36^AF680^ and monoclonal antibody HIT2^PerCP/Cy5.5^ bound to all six CD38-expressing cell lines. Pretreatment with daratumumab almost completely blocked binding of HIT2^PerCP/Cy5.5^, indicating that daratumumab and HIT2 bind to overlapping epitopes on CD38. Pretreatment with daratumumab did not block binding of JK36^AF680^, confirming independent binding of nanobody JK36 and daratumumab. Pretreatment of cells with heavy chain antibody JK36-hcAb completely blocked the binding of nanobody JK36^AF680^, confirming specific binding to human CD38.

*Ex vivo* binding of nanobody JK36^AF680^ to daratumumab-pretreated primary myeloma cells was assessed using cell suspensions from human bone marrow biopsies from patients with multiple myeloma (**Figure 3B**). The results confirmed specific labeling of daratumumab-pretreated primary myeloma cells with nanobody JK36^AF680^. Again, pretreatment of cells with heavy chain antibody JK36-hcAb completely blocked the binding of nanobody JK36^AF680^.

Fluorescence microscopy of JK36^AF680^-labeled YAC-1 CD38+ cells revealed prominent staining of the cell surface (**Figure 3C**). Pretreatment with daratumumab did not affect cell surface labeling with JK36^AF680^, while pretreatment with JK36-hcAb completely blocked binding of JK36^AF680^.

These results demonstrate that nanobody JK36^AF680^ can be used for the specific detection of daratumumab-pretreated CD38-expressing cells *in vitro* and *ex vivo* and therefore warrants further evaluation for imaging of CD38-expressing tumors *in vivo*.

### Nanobody JK36^AF680^ allows specific imaging of daratumumab-pretreated CD38-positive tumors *in vivo*

**Figure 4:**
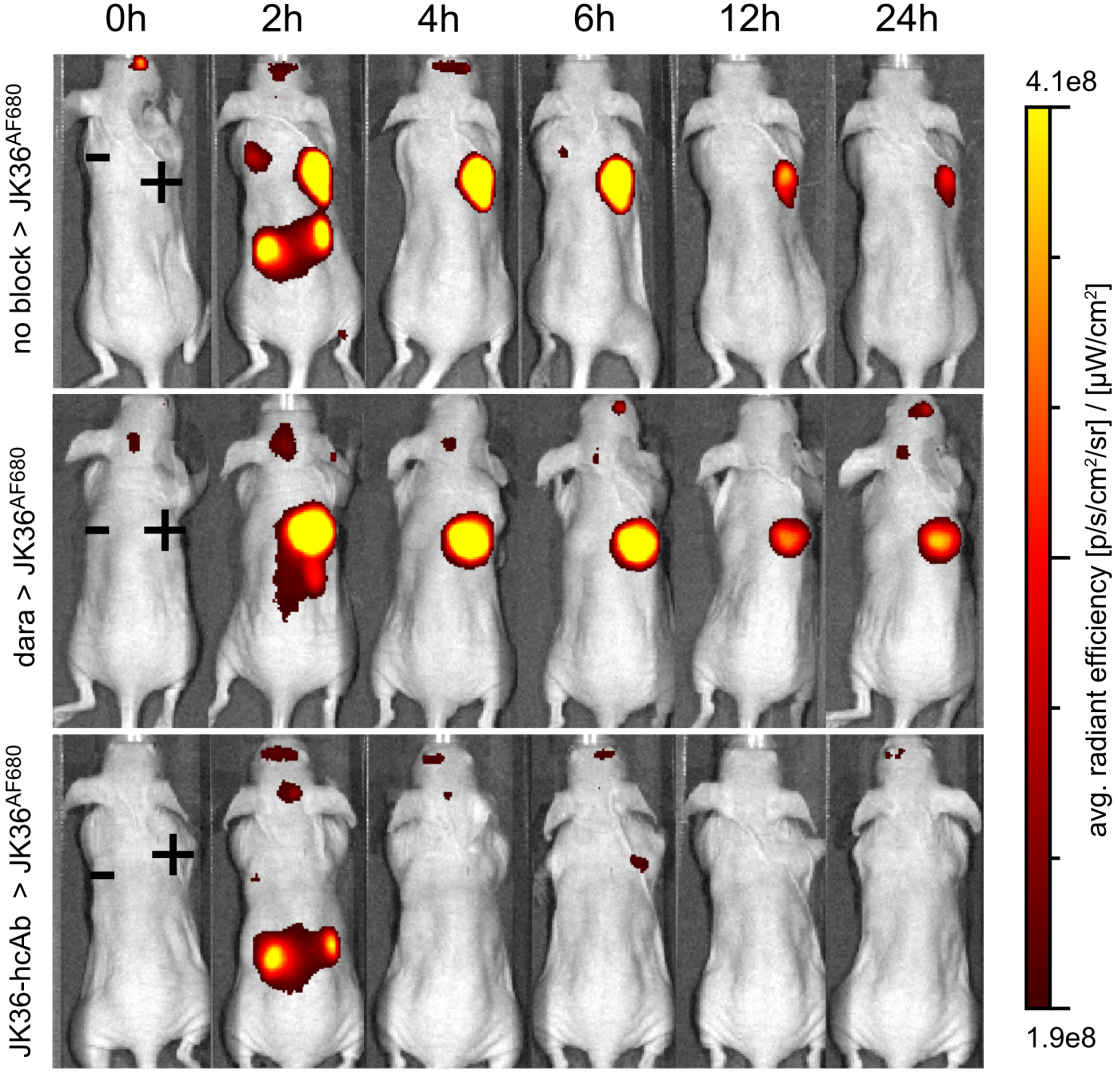
Specific *in vivo* imaging of subcutaneous CD38+ tumors using nanobody JK36^AF680^ in saline-, daratumumab-, and JK36-hcAb-pretreated mice. All mice were analyzed six days after subcutaneous injection of CD38-positive YAC-1 cells on the right shoulder (+) and CD38-negative YAC-1 cells on the left shoulder (-). Prior to imaging, mice were pretreated for 24 hours with either isotonic saline (n=10, positive control, top row), daratumumab (n=10, middle row), or JK36-hcAb (n=10, specificity control, bottom row). Near-infrared fluorescence *in vivo* imaging was performed before (0h) and at the indicated time points after the injection of 50 µg of nanobody JK36^AF680^. Signal intensities of all injected mice and imaging time points are all equally leveled to allow direct and fair visual comparison.

*In vivo* imaging experiments using nanobody JK36^AF680^ were performed in mice carrying tumors derived from subcutaneously injected YAC-1 cells. All mice were bearing two subcutaneous tumors for comparative analyses of *in vivo* imaging signals: one CD38-positive tumor on the right shoulder and one CD38-negative control tumor on the left shoulder. *In vivo* near-infrared fluorescence imaging with nanobody JK36^AF680^ was performed in mice pretreated for 24 hours with either isotonic saline (positive control), daratumumab, or JK36-hcAb (negative or specificity control) (**Figure 4**).

The results demonstrate specific binding of nanobody JK36^AF680^ to CD38-positive tumors in both saline-pretreated and daratumumab-pretreated mice as early as two hours after injection. Pretreatment of mice with JK36-hcAb resulted in a strong reduction in imaging signals from CD38-positive tumors, confirming specific binding of JK36^AF680^ to CD38 *in vivo*.

At early time points after injection of JK36^AF680^, strong signals were also observed in the kidneys in all animals, reflecting passage of nanobody JK36 through the renal filtration barrier. Additionally, CD38-negative tumors showed signal intensities slightly above background signals at early time points, likely reflecting perfusion of tumors with blood containing unbound JK36^AF680^. The unspecific signal from CD38-negative tumors and kidneys decreased over time, while signal intensities remained high in the CD38-positive tumors for six hours, confirming specific binding of JK36^AF680^ only to tumors expressing CD38.

**Figure 5:**
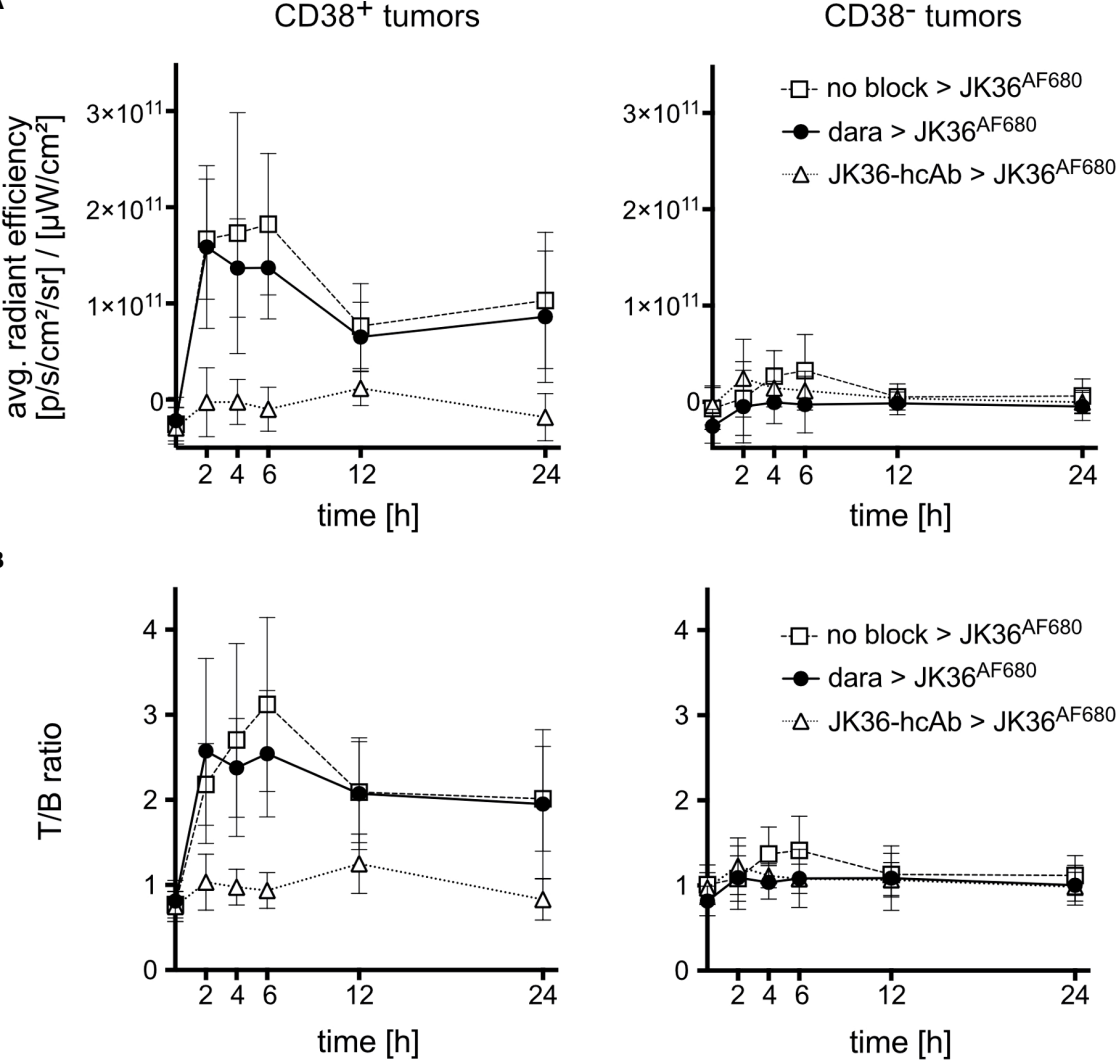
Average radiant efficiencies and tumor-to-background ratios of CD38-positive and CD38-negative tumors *in vivo*. Mice were pretreated for 24 hours with either isotonic saline (n=10, positive control, open rectangles), daratumumab (n=10, black circles), or JK36-hcAb (n=10, specificity control, open triangles). Near-infrared fluorescence *in vivo* imaging was performed before and at the indicated time points after injection of nanobody JK36^AF680^. **(A)** Average radiant efficiencies were determined from circular regions of interest (ROIs) drawn around CD38-positive and CD38-negative tumors. Signal intensities were corrected for background signal. **(B)** Tumor-to-background ratios were obtained by dividing tumor signals by background signals obtained from ROIs drawn around normal tissue (hind leg). At all time points, there was no statistically significant difference in signal intensities and T/B-ratios of CD38-positive tumors between saline-pretreated and daratumumab-pretreated mice, as indicated by overlapping 95%-confidence intervals.

ROI analyses confirmed a rapidly increasing signal in CD38-positive tumors and little if any signal in CD38-negative tumors and background tissue (**Figure 5A**). At all time points, there was no statistically significant difference in signal intensities of CD38-positive tumors between saline-pretreated and daratumumab-pretreated mice. Pretreatment with JK36-hcAb resulted in a significant decrease in CD38-positive tumor signal intensities.

Calculation of tumor-to-background ratios (T/B-ratio) confirmed a rapidly increasing T/B-ratio of CD38-positive tumors after injection of JK36^AF680^ in both saline-pretreated and daratumumab-pretreated mice (**Figure 5B**). The T/B-ratio of CD38-positive tumors reached a maximum of 3.12 ± 1.43 after six hours in saline pretreated animals, compared to 2.54 ± 1.04 in daratumumab-pretreated animals (p=0.5049). Also, there was no statistically significant difference in T/B-ratios of CD38-positive tumors between saline-pretreated and daratumumab-pretreated mice at all other time points. CD38-negative tumors revealed significantly lower T/B-ratios when compared to CD38-positive tumors in all three treatment groups, again confirming specific binding of nanobody JK36^AF680^ to human CD38.

### *Ex vivo* analyses of cells from explanted tumors

**Figure 6:**
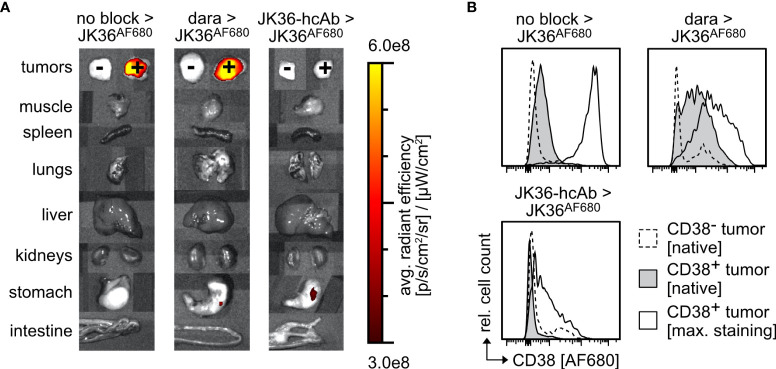
*Ex vivo* analyses of cells from CD38^+^ and CD38^-^ tumors explanted from mice 6 hours and 24 hours post-injection of JK36^AF680^. **(A)** Fluorescence levels of individual tumors and organs explanted from mice pretreated with isotonic saline, daratumumab, or JK36-hcAb 6 hours after injection of JK36^AF680^. **(B)** Histograms depicting median fluorescence intensities of the AF680 channel in mice after pretreatment with isotonic saline, daratumumab, or JK36-hcAb, obtained from tumors explanted 24 hours after injection of JK36^AF680^. Dashed histograms represent native AF680 signals of the CD38^-^ control tumor, grey histograms represent native AF680 signals of the CD38^+^ tumor, open histograms represent the maximum AF680 signal obtained after *ex vivo* staining of CD38^+^ tumor cells with JK36^AF680^. Each panel is a representative example of n=10 per treatment group. Cell debris was initially excluded by setting an appropriate FSC-A threshold before gating on single cells. YAC-1 cells were then identified by co-expression of CD38 (using nanobody JK2, identifying the remaining free epitope of CD38) and GFP.

*Ex vivo* near-infrared fluorescence imaging of dissected individual organs six hours post-injection confirmed specific uptake of JK36^AF680^ in CD38-positive tumors in both saline-pretreated and daratumumab-pretreated mice (**Figure 6A**). JK36-hcAb-pretreated animals showed no specific uptake of JK36^AF680^. Signals in most other tissues, including CD38-negative tumors, returned to background levels, with low fluorescent signals still detectable in the kidneys. While the liver itself showed only background fluorescence, fluorescent signals in the gallbladder likely reflect biliary excretion of fluorochromes.

*Ex vivo* flow cytometric analyses of CD38-positive tumors was performed six hours post-injection to quantify the relative amount of *in vivo* injected and still tumor-bound JK36^AF680^ (**Figure 6B**). Flow cytometry of tumor cells suspensions showed specific labeling of CD38-positive cells with JK36^AF680^ in both saline-pretreated and daratumumab-pretreated mice and no specific labeling of CD38-negative tumor cells. The *ex vivo* observed flow cytometric fluorescence signal obtained by *in vivo* injection of JK36^AF680^ was low in saline-pretreated and daratumumab-pretreated groups, while there was no binding of JK36^AF680^ to JK36-hcAb pretreated cells.

Additional *ex vivo* staining using JK36^AF680^ resulted in strong labeling of CD38-positive cells obtained from saline-pretreated mice. In contrast, additional *ex vivo* staining with JK36^AF680^ of daratumumab-pretreated and JK36-hcAb-pretreated mice resulted in weaker staining, possibly reflecting downregulation of CD38 due to therapeutic effects of daratumumab and JK36-hcAb *in vivo* during the 24 hours of pretreatment and six hours imaging period.

## Discussion

Our study demonstrates that a nanobody recognizing a distinct, non-overlapping epitope of CD38 allows the detection of myeloma cells pretreated with daratumumab. Nanobody JK36^AF680^ specifically detected CD38-expressing tumor cells *in vitro*, *ex vivo*, and *in vivo* irrespective of daratumumab treatment status.

JK36^AF680^ therefore represents a promising tool to overcome current clinical challenges in the assessment of treatment response in daratumumab-pretreated multiple myeloma patients (6, 10, 11). The possibility to detect and monitor CD38-expressing myeloma cells in daratumumab-pretreated patients is of increasing importance considering the increasingly broader indications for daratumumab treatment (39, 40).

Previous studies demonstrated the feasibility of fluorophore- or radionuclide-labeled daratumumab for *in vivo* imaging purposes of CD38-expressing myeloma cells (19, 20, 41). However, targeting the same epitope as daratumumab is only effective in daratumumab-naive patients. It would instead be expedient to use other labeled antibody constructs that target an epitope of CD38 distinct from that of daratumumab. This would allow not only to image untreated patients but also daratumumab-pretreated patients.

Instead of using a conventional monoclonal antibody, we used nanobody JK36^AF680^, recognizing a distinct, non-overlapping epitope of CD38 for demonstrating the feasibility of specific imaging of daratumumab-pretreated CD38-expressing tumors. Key advantages of using nanobodies over conventional antibodies for *in vivo* diagnostic purposes include greater ease of production (42) as well as more favorable imaging kinetics: nanobodies allow for excellent tumor-to-background ratios as early as 6 hours post-injection and for same-day *in vivo* imaging (33, 41), owing to the rapid elimination of excess unbound nanobodies by renal filtration. Furthermore, the risk of allergic infusion reactions is reduced when using nanobodies as opposed to conventional antibodies, owing to their low immunogenicity (43). This possibly explains the lack of infusion reactions in patients receiving caplacizumab, the first FDA-approved nanobody (44).

Additionally, a recent study revealed that our nanobody JK36 detects myeloma cells from daratumumab-treated patients with greater sensitivity than a commercially available CD38-specific multi-epitope reagent (6). Here we show that JK36 also detects CD38-expressing tumor cells with greater sensitivity than monoclonal antibody HIT2^PerCP/Cy5.5^ which is currently being used in flow cytometric assays (45–47) to assess treatment response in patients receiving daratumumab.

Isatuximab, the second FDA-approved CD38-specific monoclonal antibody, binds to a different epitope of CD38 than daratumumab (48). Further studies are warranted to evaluate the feasibility of nanobody-based imaging of multiple myeloma under isatuximab therapy, possibly using a different nanobody than JK36.

An inherent limitation of our study is the use of near-infrared fluorescent dyes for *in vivo* imaging. The signal intensity of fluorochromes such as AlexaFluor680 decreases with increasing tissue depth, precluding cross-sectional imaging of human patients (49). This limitation, however, could be overcome by coupling nanobody JK36 to a radionuclide such as ^68^Ga ^64^Cu or ^99^Tc, which would allow imaging by PET or SPECT (17, 19, 50, 51).

Since CD38 is not only expressed on myeloma cells but also on other cells (52), some level of on-target off-tissue signal is expected when using CD38-specific nanobodies for *in vivo* imaging purposes. This, however, is not a limitation of nanobody JK36 but rather a ubiquitous phenomenon when employing antibody-based imaging strategies.

In summary, we have demonstrated that a nanobody recognizing an epitope on CD38 distinct from that of daratumumab allows the specific detection of myeloma cells pretreated with daratumumab *in vitro*, *ex vivo*, and *in vivo.* Future studies using radiolabeled nanobody JK36 are warranted to investigate the potential for clinical imaging of daratumumab-pretreated human multiple myeloma patients.

## Data availability statement

The raw data supporting the conclusions of this article will be made available by the authors, without undue reservation.

## Ethics statement

The studies involving human participants were reviewed and approved by Hamburger Ärztekammer. The patients/participants provided their written informed consent to participate in this study. The animal study was reviewed and approved by Behörde für Justiz und Verbraucherschutz.

## Author contributions

PB and FK-N conceived the project. All authors carried out experiments or analyzed data. LP, PB, and FK-N wrote the manuscript. All authors contributed to the article and approved the submitted version.

## Funding

Supported by grants from the Deutsche Forschungsgemeinschaft to PB (BA 5893/7), FK-N (No. 31016 and SFB1328-Z02), and BR (SFB1328-Z02). LP was financially supported by the Else-Kröner-Fresenius-Stiftung iPRIME scholarship (2021_EKPK.10), UKE, Hamburg.

## Acknowledgments

We thank Fabienne Seyfried, Institute of Immunology, and Michael Horn, University Cancer Center Hamburg, for their excellent technical assistance. We thank the UCCH *In Vivo* Optical Imaging Core Facility and the UKE Microscopic Imaging Facility (UMIF) for their excellent support.

## Conflict of interest

FK-N receives a share of antibody sales via MediGate GmbH, a wholly-owned subsidiary of the University Medical Center Hamburg-Eppendorf. PB and FK-N are co-inventors on a patent application on CD38-specific nanobodies.

The remaining authors declare that the research was conducted in the absence of any commercial or financial relationships that could be construed as a potential conflict of interest.

## Publisher’s note

All claims expressed in this article are solely those of the authors and do not necessarily represent those of their affiliated organizations, or those of the publisher, the editors and the reviewers. Any product that may be evaluated in this article, or claim that may be made by its manufacturer, is not guaranteed or endorsed by the publisher.
